# Closed cannulation of subclavian vein vs open cut-down of cephalic vein for totally implantable venous access port (TIVAP) implantation: protocol for a systematic review and proportional meta-analysis of perioperative and postoperative complications

**DOI:** 10.1186/s13643-015-0043-1

**Published:** 2015-04-22

**Authors:** Ulla Klaiber, Kathrin Grummich, Katrin Jensen, Daniel Saure, Pietro Contin, Felix J Hüttner, Markus K Diener, Phillip Knebel

**Affiliations:** Department of General, Visceral and Transplantation Surgery, University of Heidelberg, Im Neuenheimer Feld 110, 69120 Heidelberg, Germany; Study Center of the German Surgical Society, University of Heidelberg, Im Neuenheimer Feld 110, 69120 Heidelberg, Germany; Institute of Medical Biometry and Informatics, University of Heidelberg, Im Neuenheimer Feld 305, 69120 Heidelberg, Germany

**Keywords:** Totally implantable access ports (TIAP), Totally implantable venous access ports (TIVAP), Indwelling catheters, Port catheters, Seldinger technique, Venae sectio, Open cut-down

## Abstract

**Background:**

Totally implantable venous access port (TIVAP) implantation is one of the most often performed operations in general surgery (over 100,000/year in Germany). The two main approaches for TIVAP placement are insertion into the cephalic vein through an open cut-down technique (OCD) or closed cannulation technique of the subclavian vein (CC) with Seldinger technique. Both procedures are performed with high success rates and very low complication frequencies. Because of the low incidence of complications, no single interventional trial is able to report a valid comparison of peri- and postoperative complication frequencies between both techniques. Therefore, the aim of this systematic review is to summarize evidence for peri- and postoperative complication rates in patients undergoing OCD or CC.

**Methods/Design:**

A systematic literature search will be conducted in The Cochrane Library, MEDLINE, and Embase to identify randomized controlled trials (RCTs), observational clinical studies (OCS), or case series (CS) reporting peri- and/or postoperative complications of at least one implantation technique. A *priori* defined data will be extracted from included studies, and methodological quality will be assessed. Event rates with their 95% confidence intervals will be derived taking into account the follow-up time per study by patient-months where appropriate. Pooled estimates of event rates with corresponding 95% confidence intervals will be calculated on the base of the Freeman-Tukey double arcsine transformation within a random effect model framework.

**Discussion:**

The findings of this systematic review with proportional meta-analysis will help to identify the procedure with the best benefit/risk ratio for TIVAP implantation. This may have influence on daily practice, and data may be implemented in treatment guidelines. Considering the impact of TIVAP implantation on patients’ well being together with its socioeconomic relevance, patients will benefit from evidence-based treatment and health-care costs may also be reduced.

**Systematic review registration:**

PROSPERO CRD42013005180.

## Background

**Figure 1:**
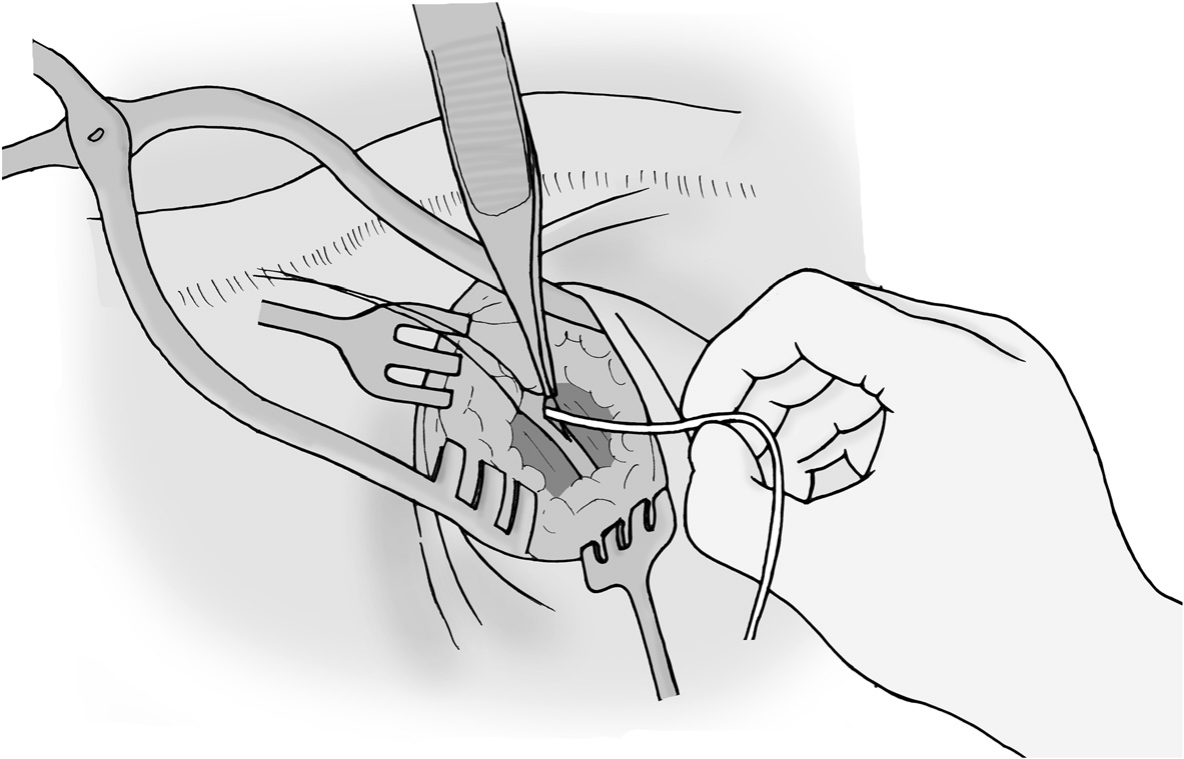
Open cut-down of cephalic vein.

**Figure 2:**
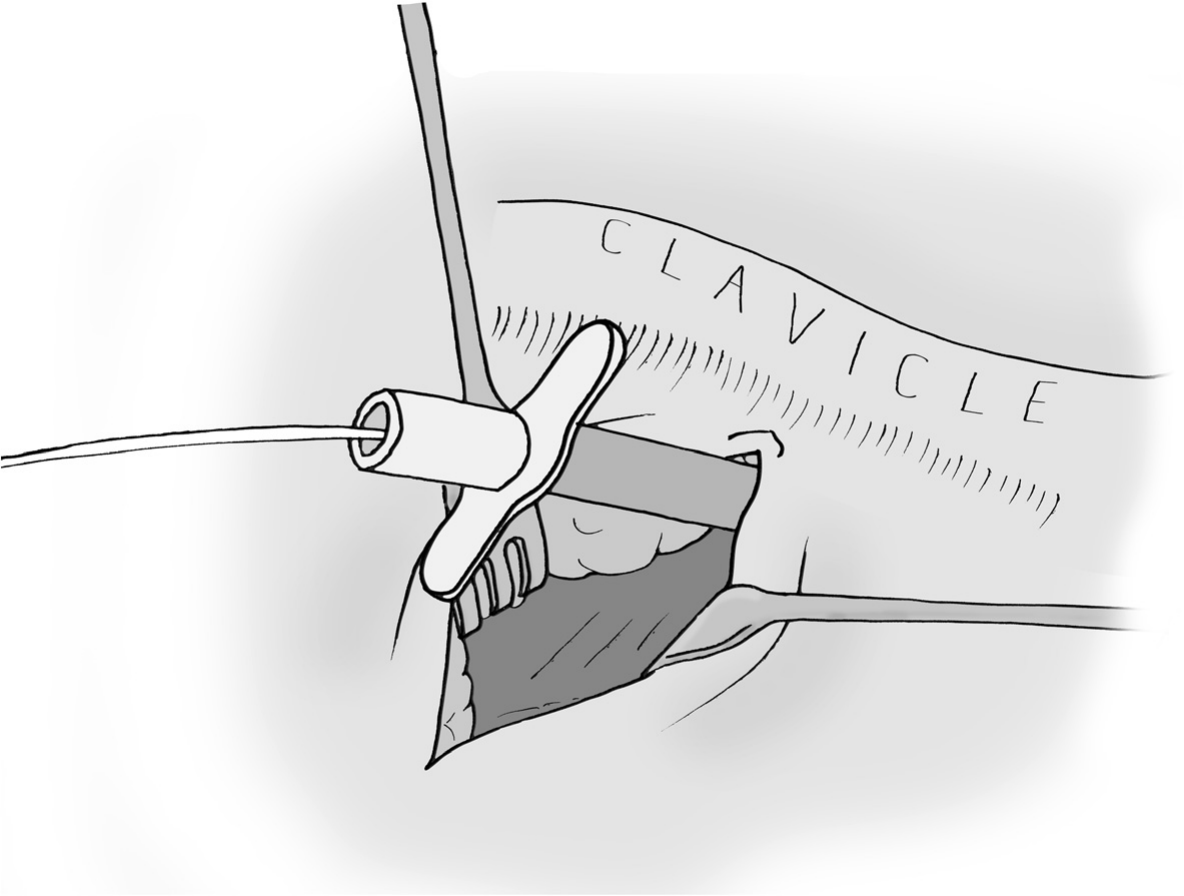
Closed cannulation of subclavian vein.

Since the introduction of totally implantable venous access ports (TIVAP) by Niederhuber *et al*. in 1982, TIVAP have been implanted routinely in patients who need a safe and permanent venous access for repeated administration of chemotherapy, parenteral nutrition, blood, antibiotics, and/or analgetics [1,2]. In daily practice, TIVAP are being extensively used worldwide and as implantation is a short procedure, it is performed preferably in an outpatient setting. In the USA, every year, approximately five million central venous catheters are inserted, whereby TIVAP are supposed to represent a considerable proportion of this number. In Germany, 477,300 new cases of oncological diseases were diagnosed in 2010 [3]. In the same year, approximately 125,790 TIVAP were performed in German hospitals and this number is constantly increasing which may be attributed to the development of innovative neoadjuvant and adjuvant oncological therapies [4]. The two main approaches for TIVAP placement are insertion into the cephalic vein through an open cut-down technique (OCD) (Figure 1) predominantly performed by surgeons with a median primary success rate of 80% (range 71% to 94%) in various prospective and retrospective trials or closed cannulation technique of the subclavian vein (CC) and insertion of the catheter with Seldinger technique (Figure 2) mainly performed by an interventional radiologist, surgeon, or anaesthesiologist with a median primary success rate of 90% to 100% in predominantly retrospective trials [5-7]. Three different techniques of CC are most common: first, CC supported by ultrasound guidance; second, CC supported by contrast agent and radiation (roadmap technique); or third, using a blind puncture method guided by anatomical landmarks (landmark technique) [2].

While common complications such as kinking or dislocation of the catheter, thrombosis, subcutaneous hematoma, and wound infection are observed in both techniques, some specific serious risks are associated only with CC, including ‘pinch off’ phenomena and pneumo- or hematothorax. The latter complications in particular require further invasive treatment, and admission to a hospital is often necessary [8]. A recent meta-analysis of six randomized controlled trials (RCTs) from 2014 showed a significantly higher primary success rate for CC but with the exclusive risk of pneumothorax occurrence [9]. In contrast, no pneumothorax was found in the OCD group.

To answer the question of overall superior benefit/risk ratio, not only the primary success rate but also peri- and postoperative complication frequencies have to be taken into account. So far, complications have been assessed as primary endpoint in only one RCT [10]. As all other trials performed to date are not powered for complications as primary endpoint, sample sizes of these studies may not be appropriate for the interpretation of peri- and postoperative complications. In most trials, low adverse event rates are presented for TIVAP implantation for both techniques. However, considering the high frequency of implantations performed, even small differences in complication rates could be highly relevant for patients and the health-care system.

None of the existing trials had a major influence on daily practice of TIVAP implantation [9]. As evidence for the strategy with the best benefit/risk ratio is poor, the choice of the technique is primarily made by the preference of the surgeon and not evidence-based. The aim of this systematic review and meta-analysis is to summarize the available evidence for peri- and postoperative complications of OCD compared to CC.

## Methods/Design

The protocol of this study is written according to the Preferred Reporting Items for Systematic Review and Meta-Analysis Protocols 2015 (PRISMA-P 2015) [11]. As outlined in this protocol, the following methods are planned.

### Systematic literature search methodology

To identify all relevant data to the scope of this review, the following searches will be performed. Published and unpublished studies investigating peri- and/or postoperative complications in TIVAP implantation performed by OCD or CC will be searched for. To answer our research question not only RCTs but also observational clinical studies (OCS) and case series (CS) will be searched for. Additionally, previous systematic as well as non-systematic reviews will be consulted. To identify all relevant studies with these characteristics, systematic literature searches will be conducted in the following databases: The Cochrane Library, MEDLINE, and Embase. For each database, an appropriate search strategy will be constructed by selecting suitable medical subject headings (MeSH) and free text terms in combination with Boolean operators. A search strategy drafted for MEDLINE is shown below. The search will not be restricted to language or status of the publication. Studies published before 1982 will not be considered as in this year; Niederhuber et al. reported the first TIVAP implantation [1]. Search results will be exported to the reference software program EndNote (version X7) to create a library in which all articles will be entered. The registries in ClinicalTrials.gov, Current Controlled Trials, and UMIN Clinical Trials Registry, as well as PROSPERO, will be searched to identify registered trials and reviews. Reference lists of relevant articles will be searched manually for additional trials. Furthermore, investigators and experts in this field will be contacted. Based on preliminary research, only very few RCTs but more than 50 OCS and CS are expected. The following is the search strategy drafted for MEDLINE:

‘port catheter’[tiab] OR ‘port catheters’[tiab] OR ‘port-a-cath’[tiab] OR ‘port-a-catheters’[tiab] OR ‘port implantation’[tiab] OR ‘port implantations’[tiab] OR TIVAP[tiab] OR TIAP[tiab] OR ‘totally implantable port’[tiab] OR ‘totally implantable ports’[tiab] OR ‘totally implantable venous access ports’[tiab] OR ‘totally implantable venous access port’[tiab] OR ‘totally implantable venous access ’[tiab] OR ‘totally implantable access port’[tiab] OR ‘totally implantable access ports’[tiab] OR ‘venous port system’[tiab] OR ‘venous port systems’[tiab] OR ‘venous access device’[tiab] OR ‘venous access devices’[tiab] OR‘ vein access’ OR ‘vein cannulation’ OR ‘vein cannulations’ OR ‘implantable injection port’[tiab] OR ‘implantable injection ports’[tiab] OR ‘subcutaneous port’[tiab] OR ‘subcutaneous ports’[tiab] OR ‘vascular access device’[tiab] OR ‘vascular access devices’[tiab] OR ‘vascular access port’[tiab] OR ‘vascular access ports’[tiab] OR ‘infusion port’[tiab] OR ‘infusion ports’[tiab] OR ‘venous cut-down technique’[tiab] OR ‘surgical cutdown’[tiab] OR ‘cutdown technique’[tiab] OR ‘seldinger technique’[tiab] OR ‘modified seldinger technique’ [tiab]

AND

complications[tiab] OR ‘wound infection’[tiab] OR ‘wound infections’[tiab] OR ‘Wound Infection’[Mesh] OR ‘Surgical Wound Infection’[Mesh] OR SSI[tiab] OR ‘surgical site infection’[tiab] OR ‘surgical site infections’[tiab] OR site infection[tiab] OR infection[tiab] OR hemothorax[tiab] OR ‘Hemothorax’[Mesh] OR hematothorax[tiab] OR haematothorax[tiab] OR pnx[tiab] OR pneumothorax[tiab] OR ‘Pneumothorax’[Mesh] OR ((dislocation OR malpositioning OR breakage) AND (TIVAP OR TIAP OR catheter OR catheters)) OR ‘nerve lesion’[tiab] OR ‘nerve lesions’[tiab] OR hematoma[tiab] OR hematomas[tiab] OR haematoma[tiab] OR haematomas[tiab] OR ‘Hematoma’[Mesh] OR ‘postoperative bleeding’[tiab] OR extravasation[tiab] OR extravasations[tiab] OR ‘Extravasation of Diagnostic and Therapeutic Materials’[Mesh] OR ‘hospital readmission’[tiab] OR ‘hospital admission’[tiab] OR ‘nerve lesion’[tiab] OR ‘nerve lesions’[tiab] OR sepsis[tiab] OR ‘septic shock’[tiab] OR ‘catheter sepsis’[tiab] OR reoperation[tiab] OR ‘TIAP explantation’[tiab] OR ‘plexus palsy’[tiab] OR ‘brachial plexus palsy’[tiab] OR ‘arterial puncture’[tiab] OR hemoptysis[tiab] OR ‘Hemoptysis’[Mesh] OR ‘pinch off’[tiab] OR ((TIAP OR TIVAP) AND Occlusion) OR ((TIAP OR TIVAP) AND (thrombosis OR ‘Thrombosis’[Mesh])) OR ‘Catheter-Related Infections/surgery’[Mesh] OR ‘Catheters, Indwelling/adverse effects’[MAJR] OR ‘Catheter-Related Infections/complications’[Mesh]

NOT

hickman OR urinary OR transfemoral OR ‘arterial infusion’ OR microcatheter OR ‘intra-arterial’ OR children OR pediat* OR paediat* OR neonate OR neonates OR emergen* OR animal OR dogs OR cats OR cadaver

### Study selection

Two independent authors will review all records identified by the abovementioned search methods. Only studies meeting the following eligibility criteria will be included: RCTs as well as prospective and retrospective OCS and CS providing data on peri- and/or postoperative complications of at least one technique for TIVAP implantation (OCD and/or CC) predominately for the treatment of underlying oncological disease in patients with at least 15 years of age will be regarded as eligible and suitable for data evaluation. Case reports, trials investigating TIVAP implantation in children, and studies focusing on patients with non-malignant diseases such as cystic fibrosis, sickle cell anemia, immunodeficiency syndrome, and other non-oncological diseases with impaired immune or coagulation system will be excluded. If the title and abstract suggest relevance, the full article will be assessed for eligibility. Any disagreements between the two reviewers will be discussed with a third reviewer to reach consent and to decide which studies to include for review.

### Data extraction

A specific sheet for data extraction will be used for the assessment of the following data from included studies: author of study, year of publication, country of publication, language, journal, study duration, study design, follow-up time and sample size. The baseline data extracted will be numbers of patients and procedures as well as participants’ age, sex, and underlying disease. Detailed information on the implantation procedures will comprise performers’ discipline, for example, surgeon or radiologist, and type of implantation technique including rescue techniques in the case of OCD and use of ultrasound, landmark technique, or fluoroscopic guidance in the case of CC. Relevant outcome variables will include perioperative complications, defined as early complications occurring during the operation or within the first 24 hours after the procedure (hemato-pneumothorax, early re-intervention due to any cause, early malfunction of TIVAP, and bleeding), as well as postoperative complications, defined as late complications emerging after the first 24 hours postoperatively (late malfunction of TIVAP, ‘pinch off’, hematoma, wound infection, late re-intervention, TIVAP infection, extravasation, TIVAP-related thrombosis, nerve lesion, TIVAP occlusion, dislocation of catheter, and hospital re-admission due to catheter problems). To check that all relevant fields have been included and to assure that the extraction of data from different study types will be feasible, the data extraction form will be piloted by extracting data from representative articles by two independent reviewers. To account for different study designs, several extraction forms will be developed, if necessary. After finalizing the document(s) data will be extracted by two independent reviewers. If there are any disagreements between the two reviewers, a third member of the working group will be consulted to discuss them.

### Assessment of the methodological quality of included studies

According to Loke *et al*. [12], the methodological quality of all studies included - independently from study design - will be assessed by means of a critical appraisal tool comprising the following three questions on the conduct of adverse effects assessment and its reporting:

1. Are peri- and/or postoperative complications the main focus of the study?
2. Are clear definitions of complications given?
3. Is the assessment of complications described in detail?

Risk of bias will be categorized as ‘low’ if all questions are answered with ‘yes’, whereas high risk of bias is assumed if all questions are answered with ‘no’. In all other cases, risk of bias is classified as ‘moderate’.

### Statistical analysis

For each perioperative outcome the event rate with its 95% confidence interval will be derived per intervention arm and per study. For each postoperative outcome, the individual follow-up time of each study is taken into account by calculating an event rate per patient-month of follow-up. This standardized event rate with its 95% confidence interval is then used per intervention arm and per study. Pooled estimates of (standardized) event rates with corresponding 95% confidence intervals will be calculated on the base of the Freeman-Tukey double arcsine transformation [13,14] within a random effect model framework. Statistical heterogeneity of combined study results will be assessed by the I^2^ statistic. Different study designs, that is, RCTs, OCS, and CS, will be analyzed separately. Within study design subgroups, additional subgroup analyses will be performed to investigate potential heterogeneity which might be caused by performers’ discipline, center’s expertise (as regards the number of implantations per month), or implantation technique (that is, CC with or without use of ultrasound, landmark technique, or fluoroscopic guidance and OCD with or without rescue techniques). Moreover, sensitivity analyses will be performed in the case of substantial differences in methodological quality of individual studies as regards the quality of adverse effects data (see above). The results will be visualized by forest plots of (standardized) event rates per intervention arm. The presence of publication bias will be explored by funnel plots with respect to the logit (standardized) event rates. Quantitative exploration of publication bias will be performed using Kendall’s tau and Egger’s regression test. For statistical analysis, the statistical software R (The R Foundation for Statistical Computing) with the ‘meta’ package (developed by G. Schwarzer) will be used.

## Discussion

TIVAP implantation is one of the surgical procedures performed most often in general surgery. The occurrence of adverse events and serious adverse events is rare for both techniques. However, considering the high number of TIVAP implantations performed worldwide, even small differences in complication frequencies between the two procedures could be of clinical relevance for patients and health-care systems. A reduction of peri- and postoperative complications may save costs for diagnostics, treatments, and hospital admissions due to complications after TIVAP implantations. Furthermore, patients may benefit from the prevention of complications as an earlier start of chemotherapy may be realized with potential impact on survival and recurrence rates of oncological diseases (for example, TIVAP-associated infections inhibit chemotherapy).

This systematic review and proportional meta-analysis are the first approach to critically appraise and quantify data on the occurrence of peri- and postoperative complications in patients undergoing OCD compared to CC for TIVAP implantation. For this purpose, all studies reporting peri- and/or postoperative complications of at least one of both techniques will be included independently of the trial design. Considering the lack of high-quality trials in surgery and the fact that case series are the most common trial design in clinical surgical research, case series will also be considered to ensure that all relevant data will be included [15]. Studies focusing predominately on patients with non-oncological diseases will be excluded as this is a minority with relatively rare underlying diseases which are often associated with specific elevated risks, for example, increased risk of infections in patients with immunodeficiency syndrome. Thus, these subgroups of patients are not considered representatives for the majority of patients in which TIVAP implantation is performed. Considering all studies focusing on patients with malignancies, the pooled sample size should be large enough to give a good overview about the distribution of peri- and postoperative complications after OCD compared to CC. Furthermore, because of the relevance and great acceptance of the two implantation techniques investigated, we expect large populations in both study groups. Thus, potential confounding variables such as treatment regimens are expected to be balanced between both groups and are not planned to be considered for data extraction.

Assessment of methodological quality will be performed according to the recommendations of Loke *et al*. [12] who recently presented a framework for a structured approach of systematic reviews of adverse effects. As to date, no appraisal tool for the assessment of methodological quality of studies included in this type of systematic review has been established; the authors advise to answer defined questions on the conduct of adverse events assessment and reporting in all studies included - independently from study design. In adherence with Loke *et al*. [12], application of critical appraisal tools commonly used for the assessment of methodological quality of studies included in systematic reviews which do not focus on adverse effects is not foreseen, because available tools such as the Cochrane Collaboration’s tool for assessing risk of bias in RCTs [16] or the Downs and Black checklist [17] may mainly apply to the primary focus of the study which is usually the beneficial effect of the intervention and not the occurrence of adverse events. Therefore, even though the methods for the assessment of the primary endpoint of the study may be of high quality, the monitoring of harmful effects of the intervention may be of low quality which may not be detected with ‘common’ critical appraisal tools.

To date, there is no gold standard for the technique of TIVAP implantation as evidence for the procedure with the best benefit/risk ratio is poor. The findings of this systematic review with proportional meta-analysis will help to compare peri- and postoperative complication rates in the two main approaches performed. This may have influence on daily practice, and data may be implemented in treatment guidelines. Considering the impact of TIVAP implantation on patients’ well being together with its socioeconomic relevance, patients will benefit from evidence-based treatment and health-care costs may also be reduced.

## Abbreviations

CC: closed cannulation technique of the subclavian vein
CS: case series
MeSH: medical subject headings
OCD: open cut-down technique
OCS: observational clinical study
PRISMA-P: Preferred Reporting Items for Systematic Review and Meta-Analysis Protocols 2015
RCT: randomized controlled trial
TIAP: totally implantable access port
TIVAP: totally implantable venous access port
